# Clarification of the internal structure and factors of poor dissolution of substandard roxithromycin tablets by near-infrared chemical imaging

**DOI:** 10.1016/j.ijpharm.2021.120232

**Published:** 2021-03-01

**Authors:** Mirai Sakuda, Naoko Yoshida, Tatsuo Koide, Tep Keila, Kazuko Kimura, Hirohito Tsuboi

**Affiliations:** a Clinical Pharmacy and Healthcare Sciences, Faculty of Pharmacy, Institute of Medical, Pharmaceutical and Health Sciences, Kanazawa University, Kanazawa 920-1192, Japan; b AI Hospital/Macro Signal Dynamics Research and Development Center, Institute of Medical, Pharmaceutical and Health Sciences, Kanazawa University, Kanazawa 920-1192, Japan; c Division of Drugs, National Institute of Health Sciences, Kawasaki 210-9501, Japan; d National Health Product Quality Control Center, Ministry of Health, Phnom Penh 12110, Cambodia; e Medi-Quality Security Institute, Graduate School of Medical Sciences, Kanazawa University, Kanazawa 920-1192, Japan

**Keywords:** Substandard and falsified medicines, Near-infrared chemical imaging, Poor dissolution, Roxithromycin, Aggregates, API, active pharmaceutical ingredient, HPLC, high-performance liquid chromatography, Mg-St, magnesium stearate, LMICs, low-middle-income countries, NIR, near infrared, NIR-CI, near infrared chemical imaging, RXM, roxithromycin, SFMs, Substandard and falsified medicines, SGDs, Sustainable Development Goals, UHC, universal health coverage, WHO, World Health Organization

## Abstract

The spread of substandard and falsified medicines has become a global problem, especially in low- and middle-income countries (LMICs). Previously, we found that some tablets containing the same active ingredient had large differences in their dissolution even though their contents were comparable. In this study, we investigated the poor dissolution of roxithromycin tablets using near-infrared chemical imaging (NIR-CI) to visualize the internal tablet structure. Roxithromycin tablets collected in LMICs and the pioneer product Rulid® as a reference were cut to a flat surface for analysis. NIR spectral data were normalized, and a principal component analysis was performed to create a tablet internal structure image. For Rulid®, the differences between the spectra with high and low scores were small, and well-defined aggregation of ingredients was not observed. However, large differences in the scores were found for roxithromycin tablets manufactured in some LMICs, and non-uniformity of ingredient distribution and aggregation were observed. Additionally, some pharmaceutical excipients, such as starch or magnesium stearate, were found in certain aggregates by comparing NIR spectra. The NIR-CI results showed some excipients existed as large aggregates, which indicated that the ingredients were not evenly mixed in the roxithromycin tablet, and this contributed to its poor dissolution.

## Graphical abstract

**Figure:**
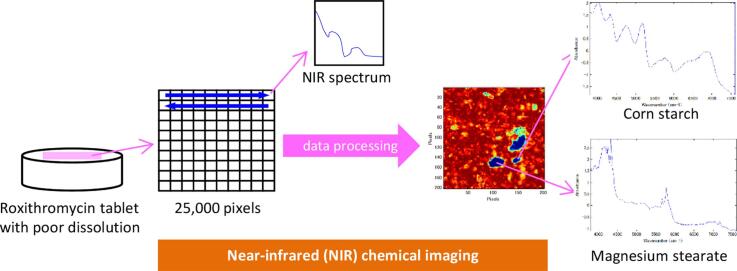

## 1 Introduction

Around the world, there are cases in which necessary medical products are not accessible. One of the major factors that contribute to the lack of access to medicines is the existence of substandard and falsified medicines. Substandard medicines, also called “out of specification medicines,” are authorized medicines that fail to meet either their quality standards, specifications, or both. Falsified medicines are defined as those that deliberately or fraudulently misrepresent their identity, composition, or source (World Health Organization, WHO, 2018). Substandard and falsified medicines (SFMs) have been found in various regions, and their existence causes not only health damage but also economic loss. This issue is exacerbated as the pharmaceutical supply chain becomes increasingly global.

It is pointed out by heads of state and the WHO that universal health coverage (UHC, Good Health and Well-being) as described in Sustainable Development Goal 3.8 (SDGs) cannot be achieved without ensuring the quality of medicines. In many countries, especially low- and middle-income countries (LMICs), medicine regulation authorities, manufacturers, and researchers are struggling to prevent the distribution of SFMs (Lon et al., 2006, Amin and Kokwaro, 2007, Gaudiano et al., 2007, Khan et al., 2010, Khan et al., 2011a, Khan et al., 2011b, Binagwaho et al., 2013, Yoshida et al., 2014, Otte et al., 2015, Petersen et al., 2017, Yong et al., 2015, Kakio et al., 2017, Khuluza et al., 2017, Kakio et al., 2018, Pisani et al., 2019, Tabernero et al., 2019, Schäfermann et al., 2020). However, the prevalence of SFMs is currently increasing globally, especially in LMICs, where the proportion of SFMs has reached 10.5% of the pharmaceutical market (WHO, 2018). No information about excipients has been disclosed in LMICs. Quality tests, especially dissolution tests, are often not conducted even though tablets with poor dissolution could result in treatment failure. In fact, in a field study in LMICs that we conducted, high rates of substandard medicines, especially poorly dissolving medicines, were detected, and it turned out that dissolution tests were often not performed (Yoshida et al., 2014). There are many factors involved in producing and distributing substandard medicines. However, there has been little progress towards identification and improvement of the processes that contribute to substandard medicines because these issues are generally attributed to the “weak technical capacity” in LMICs (WHO, 2018).

The quality testing methods for medicines commonly produced in LMICs are thin layer chromatography and a color test. Even if the content of the active pharmaceutical ingredient (API) can be confirmed using these quality methods, a dissolution test is generally not performed. The GPHF-Minilab, which is an instrument sometimes available in LMICs, can be used to perform a disintegration test, but cannot be used for a dissolution test (Petersen et al., 2017, Lalani et al., 2017). Regardless, there may be a common reason for the high frequency of the occurrence of poor dissolution of tablets in LMICs (Islam et al., 2017, Rahman et al., 2017, Sakuda et al., 2020). If the reason for the poor dissolution of tablets can be identified in the manufacturing process, we could investigate ways to improve the process by applying the latest technology, such as chemical imaging.

Chemical imaging is a concept used in many technologies to sequentially measure chemical information for each minute pixel on the sample plane and combine it with position information to form an image. When the surface of a pharmaceutical tablet is imaged by chemical imaging, the distribution of the contained ingredients is visualized. This also gives access to information about the distribution and mixing uniformity of the active ingredient and excipients in the tablet, which cannot be measured by analytical techniques, such as high-performance liquid chromatography (HPLC). Microspectroscopic chemical imaging using various spectroscopic techniques, such as infrared, near-infrared (NIR), terahertz, and raman spectroscopy, are now mainstream, but the information obtained differs depending on the method used. Chemical imaging is considered one of the best tools for understanding the manufacturing process because it gives a clear image of what is happening in the pharmaceutical product. There have been reports of using NIR chemical imaging (NIR-CI) to elucidate formulation design and process defects, as well as cases of its use in quality evaluation of medicines (Clarke, 2004, Veronin and Youan, 2004, Koide et al., 2015).

Roxithromycin (RXM) is a macrolide antibiotic derived from erythromycin. In our previous study in an LMIC in south-east Asia in 2014, the quality of the distributed RXM tablets was mostly satisfactory. Out of 57 samples of the RXM tablets collected, eight samples with an extremely low dissolution rate that did not meet the criteria of the Pharmacopoeia were found (Islam et al., 2017). It was shown that these five samples had two patterns of poor dissolution; that is, although they contained the proper amount of API, they did not disintegrate, and those that did disintegrate, did not dissolve. These findings suggested that there is more than one cause for the poor dissolution. Furthermore, we suspect that there may be problems with the pharmaceutical formulation or manufacturing process. The situation will likely be improved if the cause of the poor dissolution could be clarified, and feedback could be given to manufacturers.

In this study, we investigated the cause of the poor dissolution of RXM tablets collected in our previous study (Islam et al., 2017). The existence of aggregates has been identified as one of the causes of delayed disintegration in another previous study and thus, we hypothesized that it may contribute to the poor dissolution of RXM tablets (Fukami et al., 2016). In this study, we used NIR-CI, which has rarely been used in the evaluation of SFMs, to identify tablets with non-uniform ingredient distribution and aggregation. By extension, the results from this study may be applicable to the identification of other substandard medicines so that manufacturing processes can be investigated for ways to improve drug quality.

## 2 Materials and methods

### 2.1 Materials

RXM tablets (n = 57) manufactured by 28 different manufacturers in mostly LMICs were analyzed as the sample tablets (Table 1; Islam et al., 2017). Of these samples, 17 were found to have poor dissolution, and five of them exhibited extremely poor disintegration (Table 1). The samples were further coded based on which brand product they were as follows: product A (A044), B (B038), C (A048, B057, and B113), D (A131), and E (A049 and B086). The authentic product of the RXM tablet, Rulid® tablet 150 mg (Sanofi K.K., Tokyo, Japan), which is legally distributed in Japan, was used as the reference tablet.

**Table 1:** The background information of RMX samples.

#	Sample code	Product code*	manufacturers’ location	Dissolution**	Disintegration**
0	standard	Rulid	Japan	suitable	disintegrated
1	A002		India	poor	disintegrated
2	A006		India	suitable	disintegrated
3	A008		Cambodia	poor	disintegrated
4	A012		India	suitable	disintegrated
5	A017		Indonesia	suitable	disintegrated
6	A019		Vietnam	suitable	disintegrated
7	A020		Korea	suitable	disintegrated
8	A041		Korea	suitable	disintegrated
9	A042		Cambodia	suitable	disintegrated
10	A044	A	Cambodia	poor	poor
11	A048	C	India	poor	poor
12	A049	E	Cambodia	poor	poor
13	A053		Cambodia	poor	disintegrated
14	A056		Cambodia	suitable	disintegrated
15	A063		India	suitable	disintegrated
16	A077		India	suitable	disintegrated
17	A083		India	suitable	disintegrated
18	A093		Pakistan	poor	disintegrated
19	A100		Korea	suitable	disintegrated
20	A102		Thailand	suitable	disintegrated
21	A113		India	suitable	disintegrated
22	A115		India	suitable	disintegrated
23	A122		India	suitable	disintegrated
24	A125		India	suitable	disintegrated
25	A131	D	India	poor	poor
26	A137		Korea	suitable	disintegrated
27	A140		Cambodia	poor	disintegrated
28	B001		India	suitable	disintegrated
29	B002		Thailand	suitable	disintegrated
30	B013		Thailand	poor	disintegrated
31	B016		India	suitable	disintegrated
32	B017		India	suitable	disintegrated
33	B021		Cambodia	suitable	disintegrated
34	B030		India	suitable	disintegrated
35	B038	B	Cambodia	poor	disintegrated
36	B039		India	suitable	disintegrated
37	B042		India	suitable	disintegrated
38	B051		India	suitable	disintegrated
39	B-057	C	India	poor	disintegrated
40	B062		India	poor	disintegrated
41	B063		Vietnam	suitable	disintegrated
42	B073		India	suitable	disintegrated
43	B074		India	suitable	disintegrated
44	B086	E	Cambodia	poor	disintegrated
45	B088		India	suitable	disintegrated
46	B090		India	suitable	disintegrated
47	B092		Thailand	suitable	disintegrated
48	B094		India	suitable	disintegrated
49	B102		Korea	suitable	disintegrated
50	B107		France	suitable	disintegrated
51	B108		India	suitable	disintegrated
52	B113	C	India	poor	disintegrated
53	B118		Korea	suitable	disintegrated
54	B-126		India	suitable	disintegrated
55	C001		India	poor	disintegrated
56	C003		India	poor	poor
57	PA003		Indonesia	suitable	disintegrated

* A product code was given only for the poor dissolution samples, which were characterized for their Mg-St content and tablet hardness. Each brand has a different product code (A to E).

** Dissolution and disintegration results were obtained from the literature (Islam et al., 2017).

A reference standard of RXM was purchased from The United States Pharmacopeia Convention (Rockville, MD, USA). Magnesium stearate (Wako Pure Chemical Industries, Ltd., Osaka, Japan), corn starch (Yoshida Pharmaceutical Company Limited, Tokyo, Japan), and lactose (Nichi-Iko Pharmaceutical Co., Ltd., Toyama, Japan) were used as reference standards of the excipients.

The fatty acid analysis kit (YMC-Pack FA for long-chain/short-chain fatty acid analysis, YMC Co., Ltd., Kyoto, Japan), and the reference standard of magnesium stearate (Mg-St), and margaric acid (Nacalai Tesque, Inc., Kyoto, Japan) were used in the measurement of Mg-St content.

### 2.2 Sample preparation

A flat surface is preferable for NIR-CI. The samples of RXM tablets were trimmed by EM Trim (Leica Microsystems, Wetzlar, Germany), and sectioned to investigate the interior by NIR-CI measurements.

### 2.3 NIR-CI

The RXM tablets were measured by a NIR-CI system and performed according to the method described in a previous study (Koide et al., 2015). Spotlight400 (PerkinElmer, Inc., Japan, Yokohama, Japan), a line-scan chemical imaging system, was used to obtain the NIR spectra of the samples. Each spectrum was acquired from a 25- × 25-µm square pixel. The background scan was recorded using a gold mirror as the reflectance standard, and the sample scan was recorded at 16 cm^−1^ spectral resolution with four scans across the wavenumber range 7600–3600 cm^−1^. An area of approximately 4 mm × 4 mm (about 25,000 pixels) on the section of the tablet was measured. The NIR spectral data were normalized, and the images were generated based on the score from principal component analysis (PCA) using Isys chemical imaging software (version 5.0; Malvern Instruments, Ltd., Worcestershire, U.K.). PCA, an unsupervised method, was used to extract information from the NIR spectra because the excipients used in the sample tablets were unknown. The PCA results of the NIR spectra show the hue saturation value between red and blue in the NIR-CI. The red areas in the images represent a higher PCA score, while the blue areas represent a lower PCA score. The uniformity of the dispersion of ingredients was assessed by visual inspection. The aggregation was assessed by measuring the size of the colored area. The full NIR-CI spectra for the scanned region at selected pixel coordinates are also shown.

### 2.4 Mg-St Content and hardness measurement

The content ratio of Mg-St and the tablet hardness were measured in typical poor dissolution samples, A044, A048, A049, A131, B038, B057, B086, and B113 (Table 1). All of these samples were deemed unacceptable in the dissolution test according to Japanese Pharmacopoeia. In these measurements, a sample comprising three tablets were tested per measurement and analyzed using PCA.

The Mg-St determination was carried out by HPLC according to the application data provided by YMC Co., Ltd. (Kyoto, Japan). Using a fatty acid analysis kit, the carboxyl group of Mg-St was converted to 2-nitrophenylhydrazine, enabling highly sensitive detection in the ultraviolet (UV) and visible regions. HPLC analysis was conducted using a YMC-Pack FA (4.6 mm × 250 mm, YMC Co., Ltd., Kyoto, Japan) linked to an HPLC system (Alliance 2695, Waters Corporation, MA, USA) with a UV detector (2487, Waters Corporation, MA, USA). The column oven was kept at 35 °C. The mobile phase was a mixture of dilute hydrochloric acid solution (pH 4–5) and acetonitrile (15:85, v/v). The injection volume was 30 μL. The flow rate was 1.2 mL/min, and the detection wavelength for labeled Mg-St was 230 nm. Margaric acid was added as an internal standard. The analyses were performed in triplicate.

The hardness of the tablets was measured using a digital hardness tester (KHT-40, Fujiwara Scientific Co., Ltd., Tokyo, Japan).

### 2.5 Statistical analysis

Data were analyzed using the Japanese version of SPSS Statistics version 25 (IBM Japan, Tokyo, Japan). We used Fisher’s exact test to determine whether there was significant differences between two categories (for example, suitable dissolution and poor dissolution) and Bonferroni’s multiple *t*-test for multiple comparison of mean values. P values<0.05 were considered to be statistically significant.

## 3 Results

### 3.1 Observation of internal structure

**Fig. 1:**
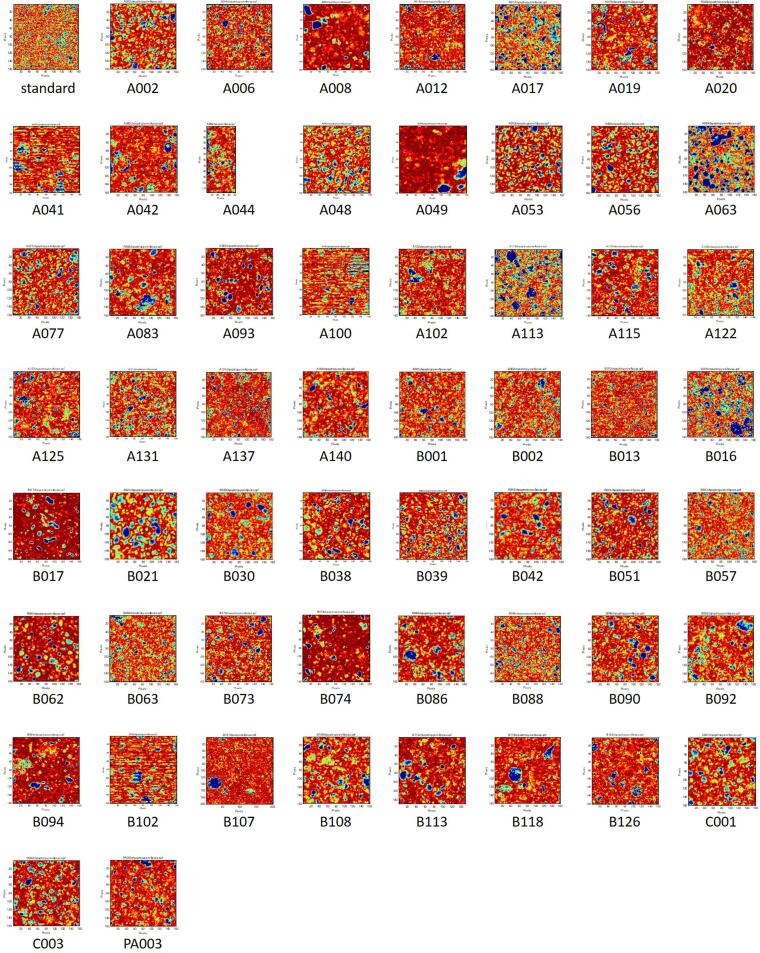
NIR-CI of all the samples analyzed.

**Fig. 2:**
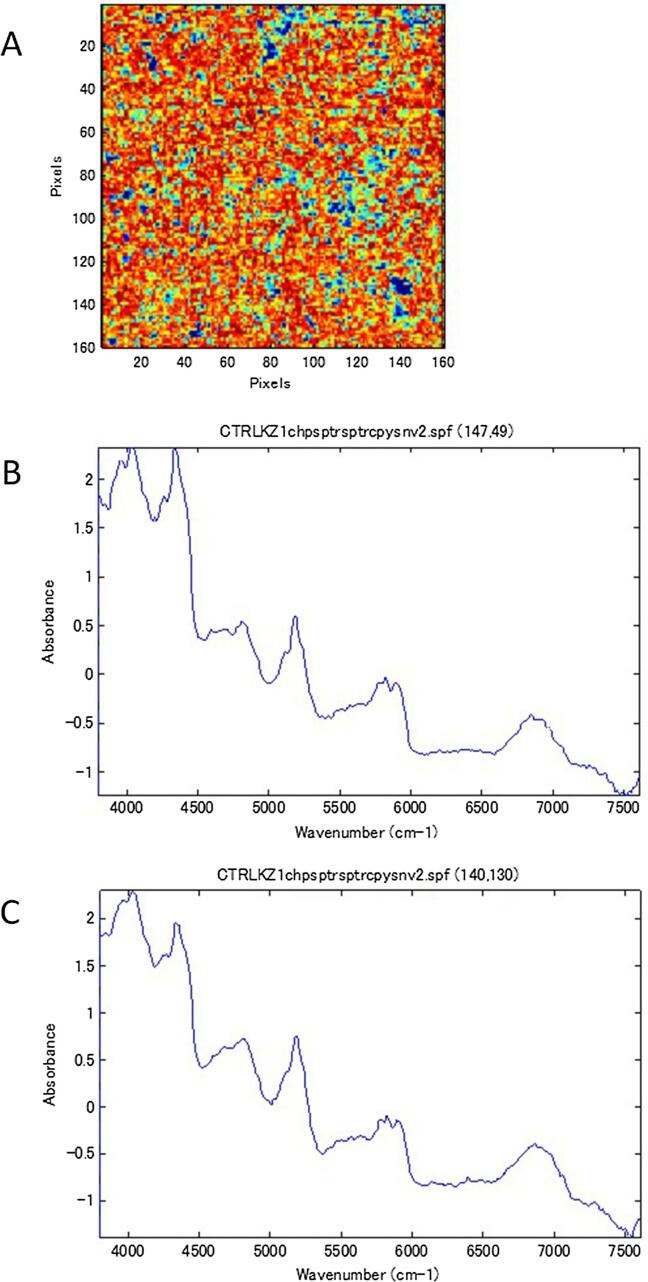
NIR-CI observed in the reference tablet (Rulid). Panel A shows the NIR-CI spectrum. Panels B and C show the NIR spectrum observed in the blue (x,y; 140, 30) and red part (x,y; 140, 50), respectively.

**Fig. 3:**
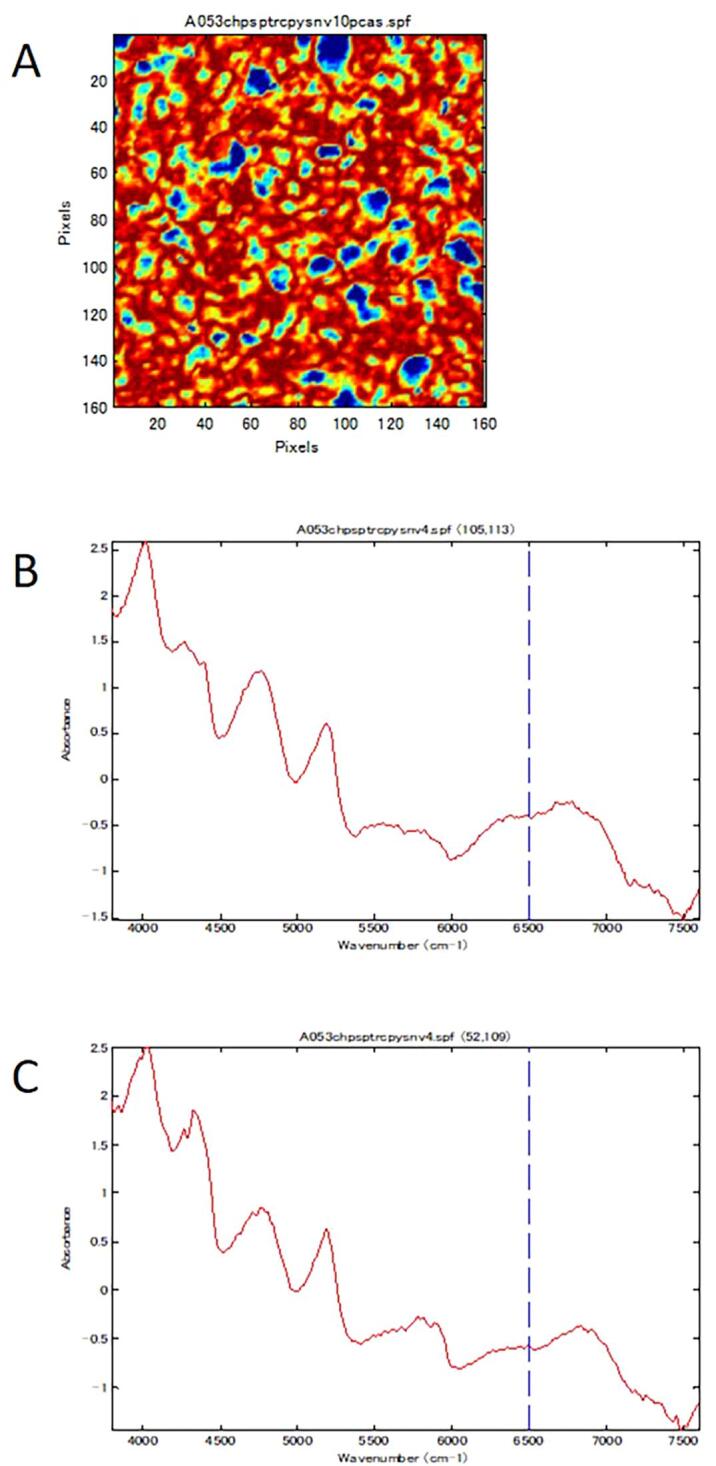
NIR-CI observed from sample A053. Panel A shows the NIR-CI. Panels B and C show the NIR spectrum observed in the blue (x,y;105, 113) and red part (x,y; 52, 109), respectively.

**Fig. 4:**
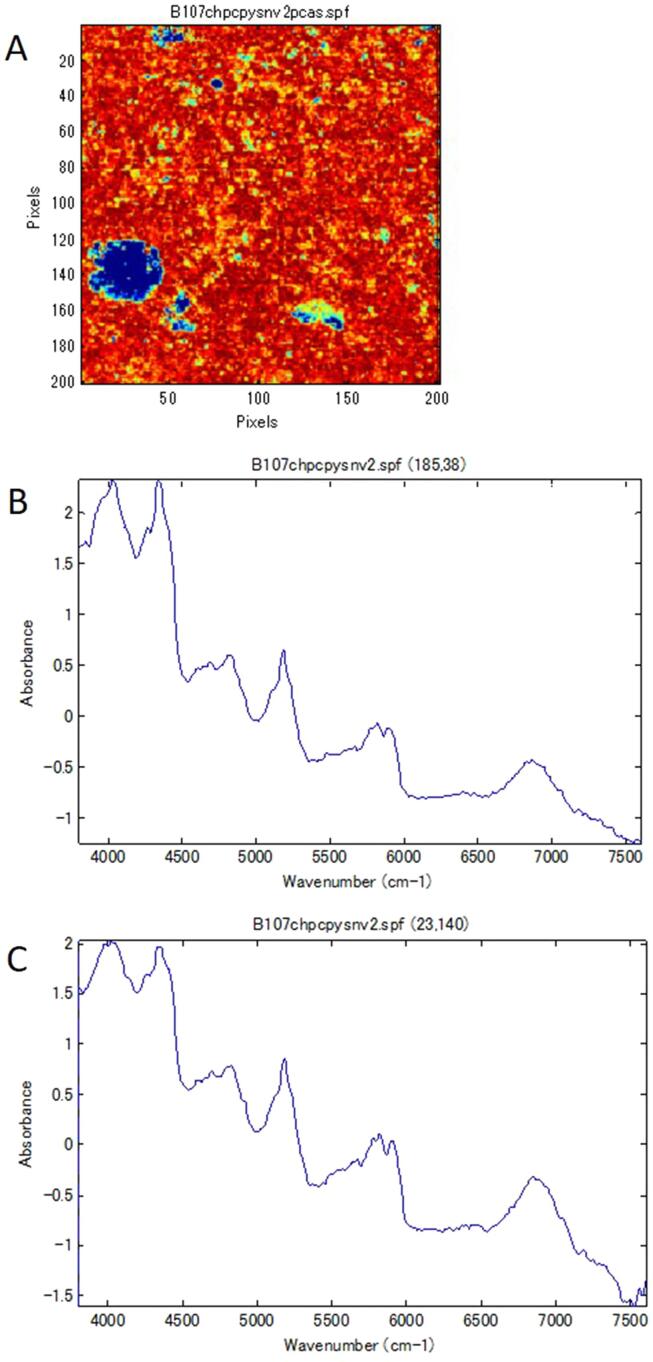
NIR-CI observed from sample B107. Panel A shows the NIR-CI spectrum. Panels B and C show the NIR spectrum observed in the blue (x,y; 231, 40) and red part (x,y; 185, 38), respectively.

NIR-CI was performed on all 57 samples of the RXM tablets and Rulid legally distributed in Japan (standard tablet). Generally, samples with images that had color aggregates with a diameter of ten pixels or more were regarded as non-uniform. Fig. 1 shows the image of the first principal component obtained from NIR-CI for all samples. In the standard tablet and three samples (A137, B013, and B063), the parts colored red or blue were smaller than ten pixels and were scattered uniformly. Aggregates larger than ten pixels appeared in the other 54 samples. However, it was also necessary to compare the NIR spectra of the aggregated parts for differences and confirm that in the samples considered to be non-uniform, large differences also existed in the NIR spectra. In the standard tablets, the difference in the NIR spectra of the high score part, colored red, and the low score part, colored blue, was small, so it was interpreted that the dispersion of the ingredients was uniform (Fig. 2). In samples A041, A100, and B102, the spectral differences between the red part and the blue part were small although the surface lines (irregularities) resulting from the carving-out sample preparation appeared in the image. Although the samples of A012, A017, A019, A020, and A053 appeared to have aggregates, almost no difference was observed between the NIR spectra of the relevant part and the other parts, and no aggregation of the ingredients was observed (Fig. 3). A large blue part was shown in a sample tablet of Rulid® (B107), but the differences in the NIR spectra between the blue part and the red part were small. This color difference was relative, and the distribution of the ingredients was interpreted as practically uniform (Fig. 4). In the samples of C001 and C003, relatively large parts of red and blue were observed (Fig. 1), but the differences in the NIR spectra were also small, and the dispersion was uniform. However, in 43 samples, large differences were observed in the spectra depending on the level of the PCA score, the ingredient dispersion was non-uniform, and it was suspected that there were some parts where the ingredients were aggregated. No relationship was observed between poor dissolution and the presence or absence of aggregates (Table 2). Although the relationship between disintegration and the presence or absence of aggregates could not be analyzed due to an insufficient number of samples, the sample in which no aggregation was observed did not show poor dissolution in this study.

**Table 2:** The relationship between dissolution or disintegration and the presence of aggregation.

		Number of tablets tested	Absence of aggregation	Presence of aggregation	*p* value (Fisher’s exact test)
dissolution	Suitable dissolution	40	5	35	0.656
	Poor dissolution	17	1	16	
disintegration	Disintegrated	52	6	46	Not applicable
	Not disintegrated	5	0	5	

### 3.2 Identification of aggregation ingredients

**Fig. 5:**
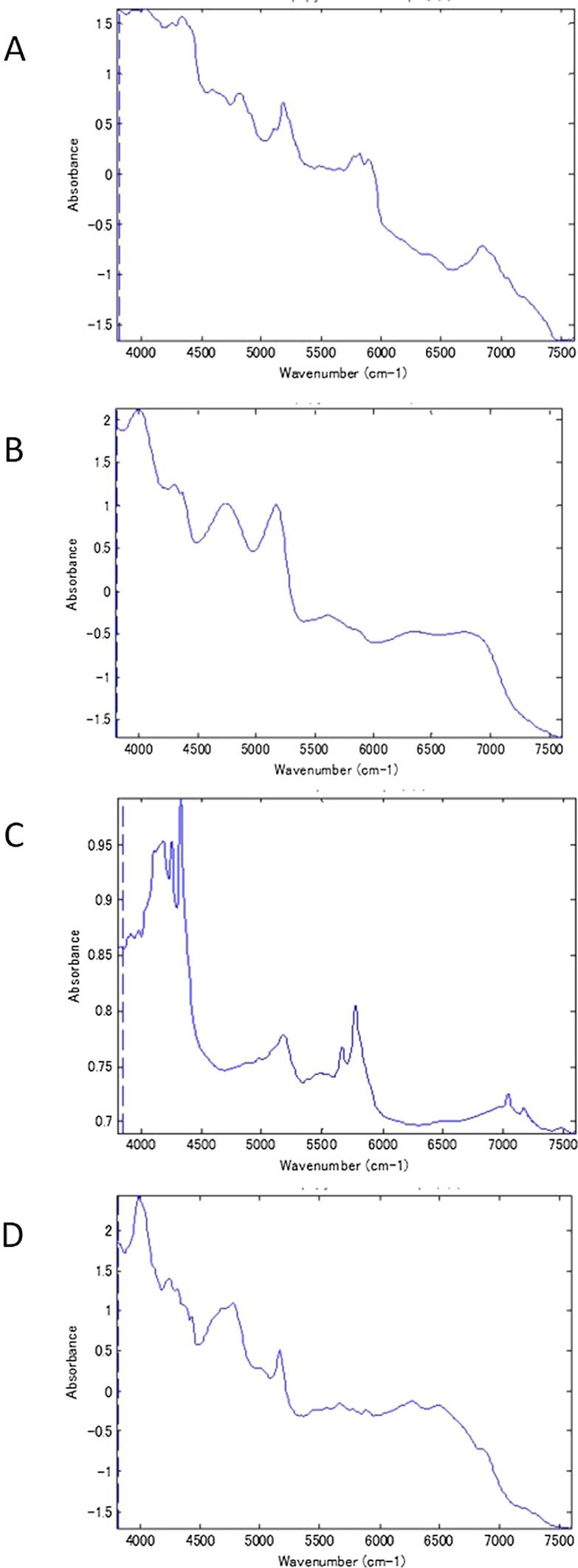
NIR spectra of the reference standard of RXM and its main excipients. Panels A, B, C, and D show the NIR spectra of the reference standard of RXM, corn starch, Mg-St, and lactose, respectively.

**Fig. 6:**
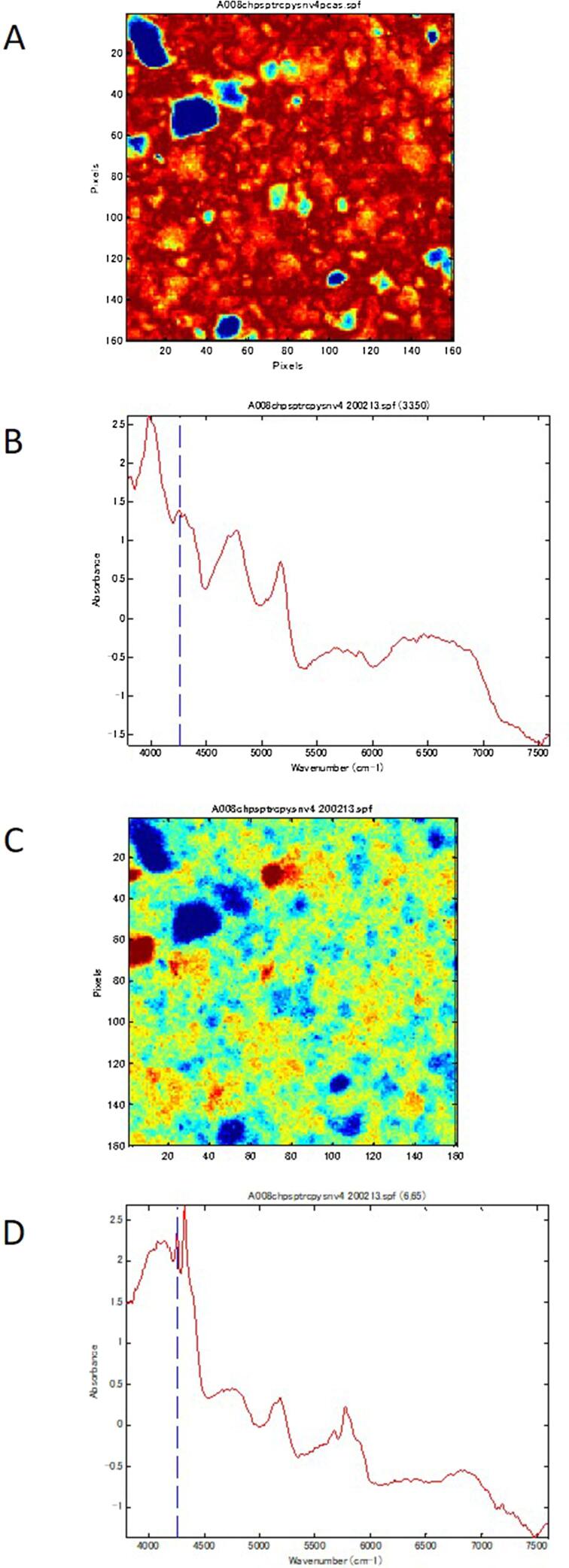
NIR-CI observed from sample A008. Panel A shows the NIR-CI. Panel B shows NIR spectra observed in the blue part (33, 50), indicating aggregation of starch. Panel C shows the image from the absorption of 4256 cm^−1^, indicating dispersion of Mg-St. Panel D shows the NIR spectra observed in the blue part (6, 65), indicating aggregation of Mg-St.

The aggregated ingredients were confirmed by comparing the NIR spectrum of the aggregated parts with the reference standard RXM spectra and the main excipients of the 43 samples in which aggregation of the ingredients was suspected. The NIR spectra of the reference standard of RXM and the main excipients, including Rulid®, are shown in Fig. 5. In A008, the NIR spectra of the aggregation parts were similar to those of starch and Mg-St. The image resulting from absorption at 4256 cm^−1^, where a peak specific to Mg-St appears, shows the dispersed state of Mg-St, where it was aggregated, and where it was dispersed (Fig. 6). In the same manner, each aggregated ingredient was confirmed. Especially in A008, A049, and B017, relatively large aggregation areas of Mg-St and corn starch were found from the NIR spectra. The most frequently found ingredients in the aggregated areas were RXM (35 samples), followed by corn starch (24 samples), Mg-St (17 samples), and lactose (5 samples). The NIR spectra of the aggregated parts found in two samples (A083 and C001) were not similar to the reference standard of RXM, corn starch, Mg-St, or lactose, and could not be identified in this study. As a result of comparing poor dissolution with the presence or absence of aggregates of RXM, corn starch, Mg-St, or lactose, a significant relationship between the existence of aggregates of corn starch and poor dissolution was shown (Table 3). By comparison to the disintegration, no relationship was observed with the aggregates of any of the ingredients (Table 4).

**Table 3:** The relationship between dissolution and the presence of aggregates of RXM, corn starch, Mg-St or lactose.

		Number of samples having aggregation				
		RXM	corn starch	Mg-St	lactose	Unknown ingredient
dissolution	Suitable dissolution (n = 40)	26	11	9	3	2
	Poor dissolution (n = 17)	9	13	8	2	1
*p* value (Fisher’s exact test)		0.553	0.001	0.111	0.629	1.000

**Table 4:** The relationship between disintegration and the presence of aggregates of RXM, corn starch, Mg-St, or lactose.

		Number of samples having aggregation				
		RXM	corn starch	Mg-St	lactose	Unknown ingredient
disintegration	Disintegrated (n = 52)	32	20	14	5	3
	Not disintegrated (n = 5)	3	4	3	0	0
*p* value (Fisher’s exact test)		1.000	0.151	0.151	Not applicable	Not applicable

### 3.3 Mg-St Content and hardness of tablets

**Fig. 7:**
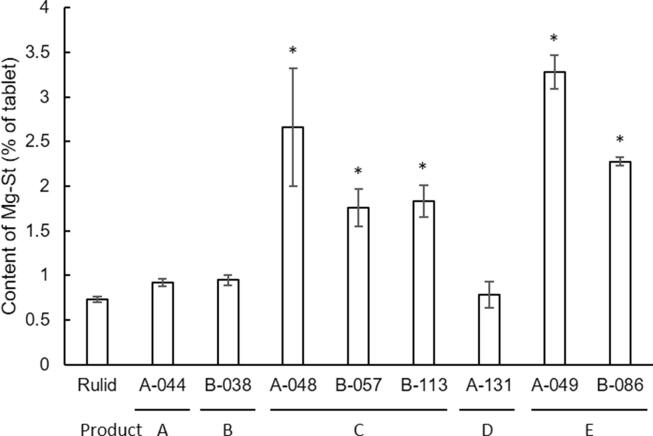
The content of Mg-St in the sample tablets. Each value represents the mean ± SD. Significant difference was compared by Bonferroni’s multiple *t*-test.

The content of Mg-St detected in eight samples (product A–E) having poor dissolution, and standard Rulid® is shown in Fig. 7. In all eight samples, the existence of aggregates was shown by NIR-CI (Fig. 1). In the NIR-CI, A044 and B113 showed the presence of aggregates of Mg-St, corn starch, and RXM, but the others showed aggregates of only corn starch and RXM. Out of eight samples, three (A048, A049, and B086) contained Mg-St amounts exceeding the typical Mg-St content of 2%. In product C (A048, B057, and B113) and product E (A049 and B086), it was shown that the content of Mg-St was not uniform even in the same product. There was no significant difference in the ratio of Mg-St content relative to the whole tablet weight in Product A (A044), B (B038), and D (A131) compared with the reference tablet, but the samples of product C (A048, B057, and B113) and product E (A049 and B086) had a significantly higher Mg-St ratio in the tablet than Rulid® (Bonferroni’s multiple *t*-test, p < 0.05).

**Fig. 8:**
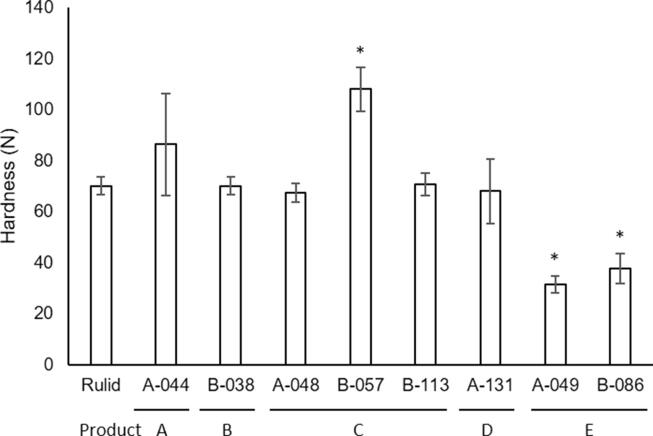
Hardness of sample tablets. Three tablets were analyzed per sample. Each value represents the mean ± SD. Significant differences were compared by Bonferroni’s multiple *t*-test.

The tablet hardness measurements are shown in Fig. 8. There was no significant difference in the hardness of the tablet in Product A (A044), B (B038), a part of C (A048 and B113), and D (A131) compared with the reference tablet, but a sample of product C (B057) had significantly higher hardness compared with the reference tablet (Bonferroni’s multiple *t*-test, p < 0.05). Particularly high tablet hardness was observed in B057, showing that the hardness of the tablets was not uniform even in the same product (product C; A048, B057, and B113). Product E (B049 and B086) had significantly lower hardness compared with the reference tablet (Bonferroni’s multiple *t*-test, p < 0.05).

## 4 Discussion

In this study, the dispersion condition of the ingredients inside RXM tablets in which poor dissolution and disintegration were found was revealed by using NIR-CI.

In the standard tablet of Rulid® and some samples of RXM tablet, the dispersion of the ingredients was uniform by visual observation, but in many samples, it was non-uniform and aggregation of the excipients and RXM was observed. From visual observation using NIR-CI, three samples (A137, B013, and B063) had uniform dispersion (Fig. 1). In the other samples, the existence of aggregation was suspected from the presence of colored regions larger than ten pixels in diameter (Fig. 1). Because the PCA score might actually maximize small differences, it was important to compare the NIR spectra of the different colored parts, especially the red and blue colored parts, to confirm whether the aggregates were actually present. In the standard tablet of Rulid, the NIR spectra were almost the same in the both of blue and red colored regions in the image, even at coordinates with different colors, suggesting there was no aggregation and the ingredients were uniformly mixed (Fig. 2). If aggregation of ten pixels in diameter or more was observed from the PCA score, but the NIR spectra at different coordinates were similar, it also suggested that there was no aggregates in the tablet (Fig. 3, Fig. 4). These results indicated that an approach to correctly interpret the resulting NIR-CI spectra across a range of wavenumbers is essential. After reviewing the data, we found 43 samples with some aggregates.

In this study, we compared the NIR spectra obtained from the colored parts in the images with those of the API and excipients and used the reference spectra to clarify whether the ingredients were aggregating in the test samples (Fig. 5). Because the actual excipients are often unknown in LMICs, we proceeded with caution. At the very least, the presence or absence of aggregation was confirmed for RXM as the API, and the excipients corn starch, Mg-St, and lactose. The distribution of API and excipients was revealed by observing the spectra at a coordinate in the image and comparing it with the characteristic spectrum of the ingredients. In one sample (A008), multiple agglutinating ingredients were found, suggesting an improper manufacturing process (Fig. 6). In the same manner, the samples with a high color contrast between the red part and the blue part (A049 and B017) had aggregates of excipients and RXM (Fig. 1).

Furthermore, the effect of the existence of aggregates on dissolution and disintegration was examined. As a result, we found that there was a significant relationship between the aggregation of corn starch, which generally acts as a disintegrant by being dispersed in the tablet, and poor dissolution (Table 3), which indicates that the existence of corn starch aggregates may be a cause of poor dissolution, although not all the aggregation sites examined in this study. However, there was no significant relationship between the aggregation of any other ingredients and poor dissolution (Table 2). There was also no significant relationship between the aggregation and poor disintegration (Table 4) in the samples tested in this study. Because the samples in which no aggregates were observed (A041, A100, A137, B013, B063, and B102) did not have poor dissolution or disintegration, aggregation might be one of the causes of poor dissolution. In the NIR analysis in the aggregated area of sample tablets, the NIR spectra of RXM, starch, Mg-St, lactose, and an unknown ingredient were identified, which indicates the possibility of unsuitable mixing or characteristics in the manufacturing process. Merely extending the mixing time may not be sufficient to improve the pharmaceutical’s quality. It is well known that lubricants such as Mg-St cause a delay in tablet disintegration/dissolution time because of excessive addition or excessive mixing. In this study, the content ratio of Mg-St might not affect the dissolution rate because it was observed that the samples with both a significantly higher and equal ratio of Mg-St compared with the standard Rulid® exhibited poor dissolution (Fig. 7). These results indicated that Mg-St content among poor dissolution samples was variable, and it is difficult to predict the existence of the aggregation from a high content of Mg-St. It was also possible that the tableting pressure increased because of the use of raw materials with large particle sizes, causing poor dissolution; however, no difference was found in the hardness of the sample tablets, so this possibility is not likely (Fig. 8). In this study, a uniform dispersion of the ingredients was shown in standard Rulid®, and a sample of Rulid® (Fig. 1, Fig. 2). Though some manufacturers, such as the innovator manufacturer, may process the particle size of the raw material, including excipients, because RXM is poorly soluble, other manufacturers may use the materials without screening and adjusting for the particle size, which may cause poor dissolution.

As for the other possibilities, the quality of the raw materials may be different and affect the final product. Moisture absorption because of poor storage conditions, such as high temperature and high humidity, might be one of the causes of quality deterioration. It has been reported that tablets contain a large amount of high specific gravity ingredients, even in falsified medicines that may be manufactured in a sloppy manner (Kakio et al., 2018). The poor dissolution in RXM tablets might be because of the manufacturing process. Considering the pharmaceutical and environmental circumstances, an appropriate overhaul of each procedure in the manufacturing process is encouraged.

Medicines play an important role in maintaining people's health, preventing illness, and saving lives. However, the presence of SFMs poses significant risks to individuals, such as lost treatment opportunities, treatment failures, and they could even be life-threatening (Rahman et al., 2018). We have also reported that poor quality antibiotics have been detected in the common pharmaceutical market in a LMIC (Yoshida et al., 2014, Islam et al., 2017, Khan et al., 2013, Rahman et al., 2019). Although most medicines were of good quality, some of these have been intentionally falsified, while others have been unintentionally degraded. Previous studies have also pointed out that improper storage and handling of medicines in tropical countries can lead to an unintentional lowering of quality (Khan et al., 2013, Newton et al., 2010). In addition, it cannot be denied that the presence of SFMs might cause improper use of antibiotics and allow resistant bacteria to be generated. RXM does not appear on the list of essential medicines. However, it is widely and commonly used in LMICs, and the medicine regulatory authorities have also been paying attention to its quality. It is necessary to guide manufacturers to improve their processes so that the medicines they produce are of satisfactory quality.

Despite the increasing proliferation of SFMs, there are few reports surmising the manufacturing method and conditions of these medicines as distributed in the pharmaceutical market. Furthermore, it is possible to propose improvement measures by applying the latest technology, such as chemical imaging technology, and clarifying the cause of the occurrence of substandard and falsified medicines and the relationship between the formulations and consideration of local pharmaceutical circumstances, such as the raw material characteristics and distribution, and manufacturing procedures; these are presented through our research. Feasible measures could become the tools for promoting a breakaway from the situation where SFMs are spreading. If a novel medicine, developed by overcoming a very low success rate and spending many years and enormous Research & Development costs, was provided as a substandard or falsified medicine, then the innovator stand to lose out economically, and the population welfare also suffers. Approaches based on applied research and accurate implementations are needed to secure a good distribution of pharmaceuticals.

In this study, samples of RXM tablets were trimmed, so the effects of the coating were not considered. It has previously been reported that the coating of tablets could be one of the causes of poor dissolution (Lopes et al., 2009, Fukami et al., 2016). In the RXM tablets analyzed in this study, samples with poor dissolution despite satisfactory disintegration were found. Therefore, the inside of the tablets was observed using NIR-CI. For analysis of NIR-CI spectra, the partial least squares method has been used to extract chemical information if the excipients are known. However, in this study, the excipients were unknown with the exception of those in the standard Rulid legally distributed in Japan. Therefore, because the ingredients of the tablets were generally unknown, PCA, which is an unsupervised method, was used in this study for analysis of the NIR spectra. However, we did detect aggregates of RXM as the API, and corn starch, Mg-St, and lactose as the excipients because we could confirm they were present in the standard Rulid. All aggregates of other ingredients would have been identified if the excipients were known. Also, the internal structure of the tablet observed in this study is only a one cut surface. Even in samples in which no aggregation was observed, there may still be aggregates in the uncut parts of the tablet (Palou et al., 2012). In addition, the tablets analyzed by NIR-CI were different from the tablets used in the dissolution test even among the same sample, so samples in which the pharmaceutical quality was variable from tablet to tablet might not correlate. The results of this study were obtained by analysis of RXM tablets collected in a limited region; however, it is not clear whether they have a common cause because many other pharmaceutical products with poor dissolution have been reported (Yoshida et al., 2014, Islam et al., 2017). In enteric-coated preparations, the lack of enteric coating has been reported as a cause of poor dissolution (Rahman et al., 2017). It is necessary to investigate the cause of poor dissolution according to the characteristics and composition of each formulation.

## 5 Conclusions

NIR-CI revealed the existence of the aggregates of excipients in the RXM tablets with poor dissolution, which suggests that ingredients were not uniformly mixed. It was also suggested that an appropriate overhaul of each procedure in the manufacturing process should be encouraged. Monitoring and guidance for improving the technical skills and the quality of raw materials could help maintain and enhance the quality levels of pharmaceuticals in circulation.

## CRediT authorship contribution statement

**Mirai Sakuda:** Conceptualization, Methodology, Validation, Formal analysis, Investigation, Data curation, Writing - original draft, Visualization. **Naoko Yoshida:** Conceptualization, Methodology, Formal analysis, Investigation, Resources, Writing - review & editing, Supervision, Project administration, Funding acquisition. **Tatsuo Koide:** Conceptualization, Methodology, Validation, Formal analysis, Investigation, Data curation, Writing - review & editing, Visualization. **Tep Keila:** Conceptualization, Methodology, Investigation, Resources, Writing - review & editing. **Kazuko Kimura:** Conceptualization, Methodology, Investigation, Resources, Writing - review & editing, Supervision, Funding acquisition. **Hirohito Tsuboi:** Formal analysis, Writing - review & editing.

## Declaration of Competing Interest

The authors declare that they have no known competing financial interests or personal relationships that could have appeared to influence the work reported in this paper.
